# Protein Kinase CK2—A Putative Target for the Therapy of Diabetes Mellitus?

**DOI:** 10.3390/ijms20184398

**Published:** 2019-09-07

**Authors:** Emmanuel Ampofo, Lisa Nalbach, Michael D. Menger, Mathias Montenarh, Claudia Götz

**Affiliations:** 1 Institute for Clinical & Experimental Surgery, Saarland University, 66424 Homburg, Germany (L.N.) (M.D.M.); 2 Medical Biochemistry and Molecular Biology, Saarland University, 66424 Homburg, Germany (M.M.) (C.G.)

**Keywords:** CK2, diabetes, β-cells, insulin

## Abstract

Since diabetes is a global epidemic, the development of novel therapeutic strategies for the treatment of this disease is of major clinical interest. Diabetes is differentiated in two types: type 1 diabetes mellitus (T1DM) and type 2 diabetes mellitus (T2DM). T1DM arises from an autoimmune destruction of insulin-producing β-cells whereas T2DM is characterized by an insulin resistance, an impaired insulin reaction of the target cells, and/or dysregulated insulin secretion. In the past, a growing number of studies have reported on the important role of the protein kinase CK2 in the regulation of the survival and endocrine function of pancreatic β-cells. In fact, inhibition of CK2 is capable of reducing cytokine-induced loss of β-cells and increases insulin expression as well as secretion by various pathways that are regulated by reversible phosphorylation of proteins. Moreover, CK2 inhibition modulates pathways that are involved in the development of diabetes and prevents signal transduction, leading to late complications such as diabetic retinopathy. Hence, targeting CK2 may represent a novel therapeutic strategy for the treatment of diabetes.

## 1. Introduction

Protein kinase CK2 is a ubiquitously expressed, constitutively active serine/threonine- and tyrosine kinase. With more than 500 substrates, CK2 is estimated to be responsible for up to 10% of the whole human phosphoproteome [1,2]. The CK2 holoenzyme is a tetramer, comprised of two catalytic α- or α’- and two non-catalytic β-subunits [3]. The α-subunits are encoded by two distinct homologous genes, CSNK2A1, which encodes CK2α [4], and CSNK2A2, which encodes CK2α’ [5]. The β-subunit is encoded by CSNK2B [6]. CK2β is not only a simple on–off regulator of the catalytic activity of CK2α but also regulates thermostability, substrate specificity, and the ability to attach and penetrate cell membranes [7,8,9,10]. CK2α and CK2β are also highly important for embryonic development. CK2α^−/−^ embryos die in mid-gestation with defects in the heart and neural tube [11]. CK2β^−/−^ mice die shortly after implantation with no signs of apoptosis, but reduced cell proliferation [12]. However, CK2α’^−/−^ knock-out mice are viable, but male mice exhibit globozoospermia [13].

It is well known that CK2 activity is elevated in most cancer cells and more importantly, CK2 inhibition leads to the induction of apoptosis in these cells [14,15]. Several studies have reported that this kinase is involved in various oncogenic signaling pathways such as the phosphoinositide 3-kinase (PI3K) and Wnt signaling [16,17]. The latter is associated with the regulation of cell development and cell differentiation [18]. Defects in Wnt signaling are often associated with tumorigenesis [18]. For instance, loss-of-function mutations for adenomatous polyposis coli (APC) result in β-catenin accumulation, and thus, promote the expression of a number of potential oncogenes [17]. CK2 phosphorylates β-catenin, which results in increased protein stability [19]. On the basis of these results and further studies that showed that this kinase regulates important cancer signaling pathways, multiple attempts have been made to develop chemotherapeutic drugs based on CK2 inhibition. These inhibitors include 4,5,6,7-tetrabromobenzotriazole (TBB), (2E)-3-(2,3,4,5-Tetrabromophenyl)-2-propenoic acid (TBCA), tetrabromo-1H-benzimidazole (DMAT), and CX-4945, also known as Silmitasertib [20,21,22]. The latter is currently the most specific CK2 inhibitor that has entered phase II clinical trials (NCT02128282).

Aside from the well-established role of CK2 in tumorgenesis, this kinase is also implicated in the regulation of other physiological processes including glucose homeostasis [23,24,25,26]. In the last few years, it has been reported that CK2 seems to play an important role in endocrine pancreatic functions because CK2 affects insulin expression at different cellular levels. Moreover, the kinase itself is regulated by glucose concentration in pancreatic β-cells [27]. Based on these findings, the present review will summarize the current knowledge about the putative function of CK2 in type 1 diabetes mellitus (T1DM) and type 2 diabetes mellitus (T2DM), the risk factors triggering T2DM as well as diabetes mellitus-associated complications.

### 1.1. CK2 Regulates Pancreatic β-Cell Death

**Figure 1:**
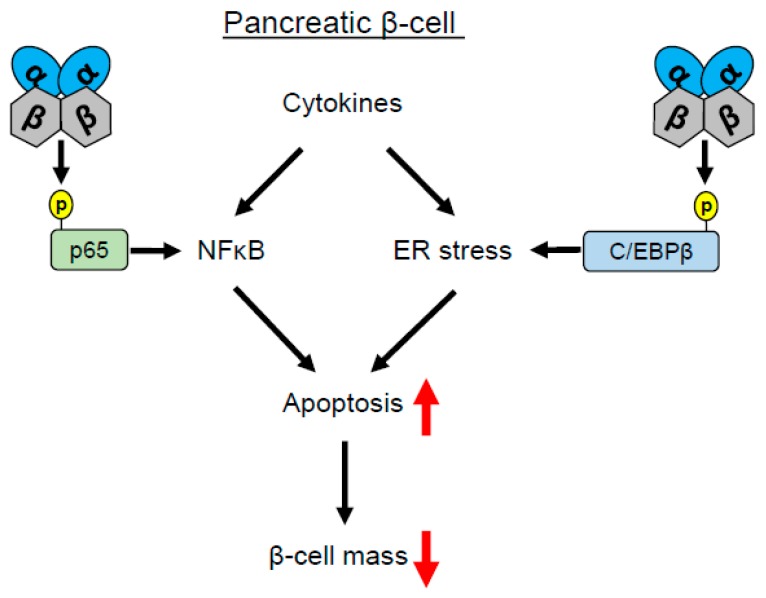
Effect of CK2 on inflammation-induced β-cell death. Cytokine-induced inflammation leads to phosphorylation of the NFκB subunit p65 as well as ER stress-induced transcription factor C/EBPβ. The phosphorylation of these proteins induces apoptotic signaling pathways, leading to a reduction of β-cell mass.

T1DM results from an autoimmune destruction of insulin-producing β-cells, leading to a complete lack or inadequate secretion of insulin [28]. In contrast, T2DM is characterized by insulin resistance, impaired insulin reaction of the target cells, and dysregulated insulin secretion [29]. Interestingly, it has also been shown that T2DM inflammation can contribute to the progression of the disease [30,31]. The inflammatory response is triggered by cytokines such as interleukin (IL)-1β, tumor necrosis factor (TNF)-α, and interferon (IFN)-γ. This leads to the induction of the pro-apoptotic NFκB pathway, resulting in a massive reduction of the β-cell mass [32]. CK2 phosphorylates the NFκB subunit p65 on serine 529 [33]. The loss of this phosphorylation results in decreased transcriptional activity and, thus, protects against cytokine-induced apoptosis [33,34,35]. Jaksch et al. [36] reported that inhibition of CK2 by DRB and DMAT reduces the re-synthesis of IκBα, which in turn inhibits the phosphorylation of p65 in β-cells. Furthermore, inhibition of CK2 results in a reduction of the IFN-α-stimulated phosphorylation of STAT1. These results indicate that the suppression of inflammatory signaling pathways by CK2 inhibition may protect β-cells from cytokine-induced cell death (Figure 1). This is quite an unusual observation, since commonly, CK2 has been shown to be a pro-survival and anti-apoptotic kinase [25,37]. On the other hand, DRB and DMAT lowered glucose-induced insulin secretion without influencing the insulin content of β-cells [36]. These results are in contrast to other studies showing that CK2 inhibition by CX-4945 markedly increases glucose-induced insulin secretion [38,39]. This could be explained by off-target effects, which may negatively affect the endocrine function of β-cells. In fact, DRB also inhibits RNA polymerase II [40], whereas DMAT induces reactive oxygen species [41].

Cytokines, released by infiltrating immune cells, disturb the endoplasmic reticulum (ER) homeostasis, which leads to ER stress during insulitis [42]. In response to these environmental changes, pancreatic β-cells trigger the unfolded protein response (UPR) by activation of ER stress sensor proteins including the transcription factor CCAAT/enhancer binding protein C/EBPβ [43]. Recently, Takai et al. [44] demonstrated that C/EBPβ accumulates in pancreatic β-cells and co-localizes with CK2 in the nucleus of β-cells following thapsigargin-induced ER stress. Moreover, the authors found a distinct, although non-canonical phosphorylation site of C/EBPβ (serine S222), which stabilizes C/EBPβ under ER stress conditions. This leads to an inhibition of AMP-activated protein kinase (AMPK)-mediated insulin secretion and an increased rate of apoptosis in pancreatic β-cells [44] (Figure 1). Aside from C/EBPβ, further CK2 substrates within the ER stress response were identified and characterized. For instance, the activating transcription factor (ATF)4 as well as the CAAT/enhancer binding protein homologous transcription factor (CHOP) are substrates of CK2 and their transcriptional activity is regulated by CK2-dependent phosphorylation [43,45,46]. These proteins are also crucially involved in diabetes, because the loss of CHOP and ATF4 results in a decline in β-cell mass, and thus contributes to the diabetic phenotype in mice [47,48]. However, further studies are required to clarify whether the CK2-dependent phosphorylation of the two proteins plays a role in ER stress-induced β-cell degeneration.

### 1.2. CK2 Regulates Insulin Expression

**Figure 2:**
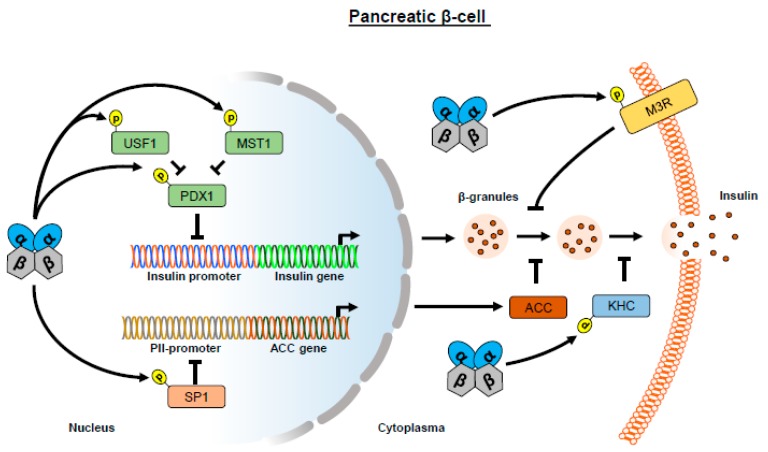
Effect of CK2 on insulin expression and secretion. CK2-dependent phosphorylation of USF1 reduces the expression of transcription factor PDX1, leading to a decreased insulin expression. CK2-induced phosphorylation of PDX1 itself also represses insulin expression by destabilizing its binding affinity to PCIF1. The kinase MST1 is also a substrate of CK2 and phosphorylates PDX1, which might also result in a decreased insulin expression. ACC induces insulin secretion by generation of malonyl-CoA. The expression of ACC is regulated by the transcription factor SP1, whose transcriptional activity is reduced by CK2-dependent phosphorylation. Hence, CK2 may repress ACC-induced insulin secretion via SP1. The muscarinic receptor M3R and KHC are both substrates of CK2 and their phosphorylation reduces insulin secretion.

A major transcription factor for pancreatic development and for the regulation of insulin expression is the pancreatic and duodenal homeobox (PDX)1 [49,50]. The relevance of this transcription factor for the development and the endocrine function of β-cells is evident in an inheritable form of diabetes, “maturity onset diabetes of the young” (MODY). We have reported that PDX-1 is phosphorylated by CK2 at serine 232 and threonine 231, resulting in a decreased insulin transcription [38,51]. In addition, these phosphorylation sites are located close to the binding site of the E3 ubiquitin ligase adapter protein PCIF1 [52,53]. PDX1 interacts with PCIF1, and this interaction is reduced by CK2 inhibition, resulting in a prolonged PDX1 half-life [54]. However, this is in contrast to the results of Ostertag et al. [55], demonstrating that CK2-dependent phosphorylation of PDX1 diminishes the binding affinity to PCIF1. CK2 and PDX1 are located in the cytoplasm under low glucose conditions. With increasing glucose concentration, the two proteins migrate into the cell nucleus [51]. Interestingly, the biosynthesis of PDX1 is regulated by upstream stimulatory factors (USF) as well as PDX1 itself by an auto-regulatory loop [56]. USF1 and USF2 belong to the basic helix loop helix (bHLH) leucine zipper family and are capable of forming the USF1/USF2 heterodimer. Recently, we found that PDX1 and USF1 interact functionally at the PDX1 promoter where USF1 acts as a transcriptional repressor [39]. Moreover, USF1 is phosphorylated at threonine 100 by CK2 [57] and the loss of this phosphorylation increases the transcriptional activity of the PDX1/USF1 complex [39], indicating that CK2 acts as a negative regulator of the auto-regulatory loop. PDX-1 is not only a substrate for CK2, but also for a number of other kinases [58,59]. Mammalian sterile 20-like kinase 1 (MST1) is a pro-apoptotic kinase that is responsive to cell stress, for instance, in a diabetic milieu. MST1 phosphorylates PDX1 at threonine 11, and thus reduces its stability [60,61]. Recently, MST1 was also identified as a substrate of CK2 [62]. Therefore, it is tempting to speculate that this phosphorylation also influences PDX1-induced insulin expression in pancreatic β-cells. These results demonstrate that CK2 indirectly suppresses insulin expression (Figure 2). Hence, CK2 inhibition represents a promising approach to improve the endocrine function of pancreatic β-cells.

### 1.3. CK2 Regulates Insulin Release

Glucose-stimulated insulin secretion (GSIS) is characterized by an increased glucose uptake into pancreatic β-cells via the glucose transporter (GLUT)1 or GLUT2. This leads to closing of the K^+^ channels, membrane depolarization, and the subsequent opening of plasma membrane associated Ca^2+^ channels. The elevated level of cytosolic Ca^2+^ ions is important for β-granule transport and insulin release [63]. Kinesin heavy chain (KHC) promotes the transport of β-granules to the plasma membrane [64]. It has been shown that CK2 phosphorylates KHC under a low level of Ca^2+^ ions. Upon the increase in the Ca^2+^ concentration, KHC is rapidly dephosphorylated by protein phosphatase 2B (PP2B). Of note, PP2B inhibition leads to deteriorated insulin secretion [65], indicating that CK2 activity represses insulin secretion by KHC phosphorylation.

Insulin secretion is also mediated by the major parasympathetic neurotransmitter acetylcholine, which acts in part through G-protein coupled muscarinic M3 receptors (M3R) [66]. Several studies have reported that the activity of M3R is regulated by different protein kinases including G-protein-coupled receptor (GPCR) kinases, CK1, and CK2 [67,68,69,70]. Rossi et al. [71] investigated the role of CK2 in M3R-mediated insulin secretion in detail and showed that CK2 phosphorylates M3R in pancreatic β-cells and that loss of M3R phosphorylation ameliorates M3R-stimulated insulin release in vitro as well as in vivo. This is in line with other studies showing that inhibition of CK2 by CX-4945 elevates GSIS [38,39]. The analyses of the underlying mechanism revealed that this was due to the activation of the protein kinase (PK)C and the phospho lipase (PL)C, resulting in an increased cytosolic Ca^2+^ concentration, which triggers the secretion of insulin from β-granules [71]. In addition, inhibition of CK2 protects from glucolipotoxicity, which is characteristic for T2DM and caused by the continued exposure of β-cells to high glucose and lipids [72]. These findings clearly demonstrate a possible therapeutic use of CK2 inhibitors for the treatment of T2DM.

Acetyl-CoA carboxylase (ACC) was proposed as one of the key elements in GSIS [73,74]. ACC is the regulatory enzyme of the fatty acid synthesis pathway, generating malonyl-CoA from acetyl-CoA. Malonyl-CoA represents the starter molecule for fatty acid synthesis and, moreover, inhibits the transport of fatty acids into mitochondria. Accordingly, long chain fatty acids accumulate in the cytosol and are capable of triggering insulin secretion [75]. The expression of ACC is under the control of the PII-promoter, which contains binding sites for the transcription factor SP1 [76]. In addition, Armstrong et al. [77] reported that CK2-mediated phosphorylation of the C-terminus of Sp1 decreased its transcriptional activity. In fact, overexpression of CK2 partially inhibits the activity of the PII-promoter [76], which may have implications for GSIS. In summary, CK2 influences insulin secretion on different levels and active CK2 acts as a molecular repressor of insulin secretion (Figure 2). Accordingly, CK2 inhibitors may be suitable for the treatment of T2DM by the amelioration of insulin secretion.

### 1.4. CK2 Regulates Insulin Signaling in Adipocytes/Fat Tissue

Obesity is a worldwide health problem that is strongly associated with T2DM. This disease is due to an abnormal accumulation of adipose tissue, resulting from chronic over-nutrition and reduced physical activity. Adipose tissue serves as a fuel storage depot, but also plays a crucial role in energy homeostasis, appetite regulation, and glucose metabolism. The elevated accumulation of fat tissue caused by adipocyte hyperplasia/hypertrophy is associated with perturbations including fatty acid secretion and dysregulated adipocyte hormone signaling [78].

Insulin is a potent adipogenic hormone that triggers the differentiation of preadipocytes into mature adipocytes (hyperplasia). Many studies have demonstrated that a sequential activation of transcription factors such as C/EBPβ, C/EBPα, and peroxisome proliferator-activated receptor (PPAR)γ2 leads to the removal of pre-adipocytes from the cell cycle and the induction of highly specific proteins like GLUT4 [79,80,81]. We have previously shown that CK2 is required for the process of adipogenesis because inhibition of the kinase within the early phase of differentiation suppresses the development of mature adipocytes [23]. The analysis of the underlying signaling cascade revealed a decreased expression of C/EBPα and PPARγ2 [82]. Moreover, Chen et al. [83] reported the vital role of deacetylase sirtuin 6 (SIRT6) in adipogenesis through the regulation of CK2 activity. They found that SIRT6 reduced the expression of kinesin heavy chain isoform (KIF)5C, which is a negative regulator of mitotic clonal expansion. KIF5C is a binding partner of CK2α’ and a substrate for CK2 [84,85]. The reduction of KIF5C expression results in a nuclear translocation of CK2α’, and thus the induction of adipogenesis [83]. Of note, CK2α phosphorylates SIRT6 in cancer cells and the loss of the phosphorylation reduces the activity of the metallopeptidase (MP)9- and β-catenin–related signaling pathways, which play an important role in tumorigenesis [86]. Therefore, it is conceivable that the activity of SIRT6 as well as KIF5C is also regulated by CK2 in adipocytes. Taken together, these data demonstrate that CK2 inhibition suppresses hyperplasia of fat tissue, and hence may protect against obesity-induced diabetes.

Besides specific transcription factors related to adipogenesis, the excess storage of triglycerides (adipocyte hypertrophy) is also regulated by CK2. Under physiological conditions, insulin promotes adipocyte glucose-uptake by plasma membrane localization of GLUT4, and in parallel, acts as an anti-lipolytic hormone by the inhibition of the hormone-sensitive lipase (HSL). Borgo et al. [87] reported that the inhibition of CK2 diminishes Akt activity, which, in turn, activates the phosphatase and tensin homolog (PTEN), resulting in a disturbed GLUT4 translocation to the cell surface. Additional in vivo analysis demonstrated that an acute pre-treatment of mice with CX-4945 suppresses glucose-uptake in white adipose tissue [87]. Of note, CK2 is upregulated in the abdominal fat tissue of human obese patients independently of insulin resistance [87]. Moreover, adipocytes secrete numerous adipokines such as leptin, resistin, and adiponectin [88]. The latter one binds to the adiponectin receptors (AdipoR)1 and 2, which are widely expressed in the tissue of the whole body including pancreatic β-cells. Several studies have shown that adiponectin stimulates insulin secretion of β-cells and, more importantly, that high plasma adiponectin levels correlate with improved insulin sensitivity, reduced inflammation, and enhanced survival of β-cells [89,90]. In 2008, Heiker et al. [91] identified AdipoR1 as an intracellular interacting protein of CK2β and also showed that inhibition of CK2 by DMAT leads to a decrease of ACC phosphorylation when stimulated with adiponectin. In a follow-up study, they found that aside from the regulatory CK2β subunit, the catalytic CK2α subunit also interacts with AdipoR1 [92]. These data demonstrate that the activity of CK2 is required for fatty acid synthesis, however, further studies are required to identify the underlying molecular mechanisms. Taken together, obesity-related diseases such as T2DM have become more and more a problem for Western civilization. Therefore, downregulation of CK2 might be a promising therapeutic approach to counteract human obesity, and thus, the development of T2DM.

### 1.5. CK2 and Diabetic Retinopathy

Increased levels of glucose in diabetic patients are thought to be a risk factor for microvascular and macrovascular complications. Diabetic retinopathy is the most common vascular complication that occurs in up to 20% of patients with diabetes and may lead to blindness in working-age adults [93]. The metabolic dysregulation results in non-perfusion and subsequent ischemia of retinal tissue. This, in turn, triggers pro-angiogenic factors, resulting in vascular cell proliferation, and thus neovascularization of retinal vasculature [94,95]. CK2 stimulates various pathways linked to angiogenesis including Ras-Raf-MEK-ERK, p38 MAPK, PKC, and PI3K-Akt [96,97]. Accordingly, inhibition of CK2 seems to be a potential anti-angiogenic therapeutic approach [24]. In fact, inhibition of this kinase reduces the vascularization of developing endometriotic lesions [98]. Moreover, treatment with CX-4945 reduces the phosphorylation of Akt and PTEN in endothelial cells, leading to a diminished microvascular tube formation [99].

Retinal endothelial cells express insulin-like growth factor receptor binding protein 3 (IGFBP-3), which is responsible for the delivery of insulin-like growth factor 1 (IGF-1) to the cells under physiological conditions [100]. In diabetic retinopathy, the expression of IGFBP-3 is markedly reduced due to elevated levels of TNF-α [101]. Of note, it has been shown that TNF-α knockout mice failed to develop diabetic retinopathy, indicating a major role of this inflammatory mediator in this disease [102,103]. Molecular analysis revealed that high glucose-induced TNF-α secretion leads to the phosphorylation of p38 and increased activity of CK2, which in turn attenuates IGFBP-3 expression [104]. Therefore, CK2 inhibition may be a suitable therapeutic strategy to suppress diabetes-induced neovascularization of retinal tissue. Indeed, Ljubimov et al. [105] demonstrated that inhibition of CK2 activity decreased normal and diabetic retina endothelial cell proliferation, migration, and viability in vitro. Moreover, pretreatment of mice with the CK2 inhibitors TBB or emodin reduced retinal neovascularization in a mouse model of oxygen-induced retinopathy [105]. In a follow-up study, they reported that CK2 was highly expressed in astrocytes near superficial retinal blood vessels during intraretinal neovascularization, whereas other cells only expressed this kinase at low levels [106]. Therefore, it is tempting to speculate that the CK2 inhibitor blocks the kinase activity primarily in astrocytes, and thus may inhibit intraretinal neovascularization.

## 2. Conclusions and Future Perspectives

**Figure 3:**
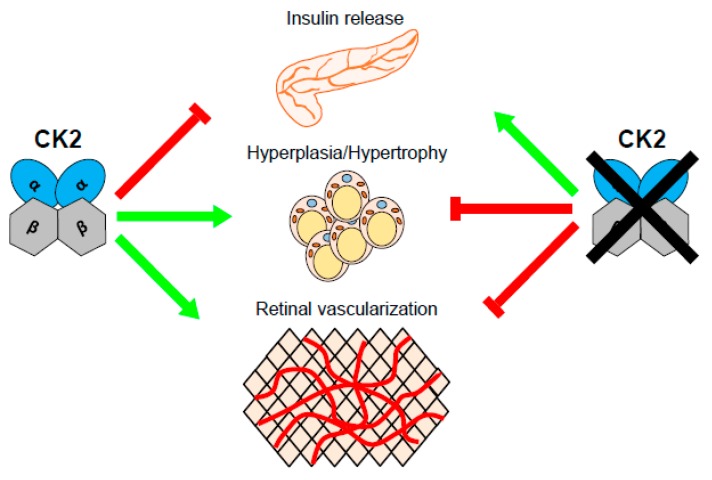
Effect of CK2 on insulin release, adipocyte hyperplasia/hypertrophy, and retinal vascularization. The protein kinase CK2 decreases the insulin release of β-cells. On the other hand, this kinase increases adipocyte hyperplasia/hypertrophy as well as retinal vascularization. Therefore, inhibition of this kinase may represent a promising therapeutic approach for the treatment of T2DM.

CK2 is ubiquitously expressed and plays an important role in many physiological processes such as thrombosis, differentiation, and cell cycle regulation [23,24,25,107]. In β-cells, it has been reported that this kinase promotes cytokine-induced cell death [36] and decreases the endocrine function [38]. The latter is achieved by the phosphorylation of proteins that are directly or indirectly implicated in the downregulation of insulin expression and secretion. Therefore, the development of anti-diabetic drugs targeting CK2 activity may be an interesting approach, as CK2 inhibition (i) increases insulin expression and secretion; (ii) reduces adipocyte hyperplasia/hypertrophy, which may counteract obesity-induced development of T2DM; and (iii) deceases diabetes-induced neovascularization of retinal tissue (Figure 3).

It has been reported that Wnt signaling impacts pancreatic β-cell function [108]. For instance, Rulifson et al. [109] demonstrated that expression of a constitutively active β-catenin in the mouse pancreas ameliorates insulin secretion. Based on this, it would be conceivable that the increased insulin secretion after CK2 inhibition may be triggered by Wnt signaling because CK2 influences Wnt signaling on multiple levels. However, downregulation of CK2 results in the subsequent degradation of β-catenin by the proteasome [19]. In contrast, blockade of Wnt signaling with a specific inhibitor of the Wnt pathway ameliorates retinal inflammation, vascular leakage, and retinal neovascularization [110]. Therefore, the inhibitory effect of CK2 inhibition on the neovascularization of retinal tissue could be due to the repression of Wnt signaling.

The currently available CK2 inhibitors have a high cell membrane penetrative capacity without cell specificity. Hence, the application of these molecules bears the risk of major side effects due to the importance of CK2 in many other processes such as cell differentiation and cell proliferation under physiological conditions. Therefore, the development of cell specific CK2 inhibitors is one of the essential factors in achieving this goal. Moreover, nothing is known about the expression, activity, and substrates of CK2 in other endocrine pancreatic cells such as α-cells thus far. These cells release the insulin-antagonist glucagon during hypoglycemia, which stimulates glucose output from the liver. Therefore, it is conceivable that CK2 inhibition may decrease glucagon expression/secretion in α-cells as a consequence of the increased insulin expression/secretion in β-cells.

## References

[1] Salvi M., Sarno S., Cesaro L., Nakamura H., Pinna L.A. (2009). Extraordinary pleiotropy of protein kinase CK2 revealed by weblogo phosphoproteome analysis. Biochim. Biophys. Acta.

[2] Nunez de Villavicencio-Diaz T., Rabalski A.J., Litchfield D.W. (2017). Protein Kinase CK2: Intricate Relationships within Regulatory Cellular Networks. Pharmaceuticals.

[3] Boldyreff B., Meggio F., Pinna L.A., Issinger O.G. (1994). Protein kinase CK2 structure-function relationship: Effects of the á subunit on reconstitution and activity. Cell. Mol. Biol. Res..

[4] Wirkner U., Voss H., Lichter P., Ansorge W., Pyerin W. (1994). The human gene (CSNK2A1) coding for the casein kinase II subunit alpha is located on chromosome 20 and contains tandemly arranged Alu repeats. Genomics.

[5] Ackermann K., Neidhart T., Gerber J., Waxmann A., Pyerin W. (2005). The catalytic subunit alpha’ gene of human protein kinase CK2 (CSNK2A2): genomic organization, promoter identification and determination of Ets1 as a key regulator. Mol. Cell Biochem..

[6] Albertella M.R., Jones H., Thomson W., Olavesen M.G., Neville M., Campbell R.D. (1996). Localisation of eight additional genes in the human major histocompatibility complex, including the gene encoding the casein kinase II beta subunit, and DNA sequence analysis of the class III region. DNA Seq..

[7] Raaf J., Brunstein E., Issinger O.G., Niefind K. (2008). The interaction of CK2alpha and CK2beta, the subunits of protein kinase CK2, requires CK2beta in a preformed conformation and is enthalpically driven. Protein Sci..

[8] Meggio F., Boldyreff B.S., Marin O., Pinna L.A., Issinger O.G. (1992). CK2: Role of the á- subunit on the stability and specificity of the recombinant reconstituted holoenzyme. Eur. J. Biochem..

[9] Boldyreff B.S., Meggio F., Pinna L.A., Issinger O.G. (1992). Casein kinase-2 structure- function relationship: Creation of a set of mutants of the á subunit that variably surrogate the wildtype á subunit function. Biochem. Biophys. Res. Commun..

[10] Rodriguez F.A., Contreras C., Bolanos-Garcia V., Allende J.E. (2008). Protein kinase CK2 as an ectokinase: The role of the regulatory CK2β subunit. Proc. Natl. Acad. Sci. USA.

[11] Lou D.Y., Dominguez I., Toselli P., Landesman-Bollag E., O’Brien C., Seldin D.C. (2008). The alpha catalytic subunit of protein kinase CK2 is required for mouse embryonic development. Mol. Cell Biol..

[12] Buchou T., Vernet M., Blond O., Jensen H.H., Pointu H., Olsen B.B., Cochet C., Issinger O.G., Boldyreff B. (2003). Disruption of the regulatory á subunit of protein kinase CK2 in mice leads to a cell-autonomous defect and early embryonic lethality. Mol. Cell. Biol..

[13] Xu X., Toselli P.A., Russell L.D., Seldin D.C. (1999). Globozoospermia in mice lacking the casein kinase II à’ catalytic subunit. Nat. Genet..

[14] Intemann J., Saidu N.E.B., Schwind L., Montenarh M. (2014). ER stress signaling in ARPE-19 cells after inhibition of protein kinase CK2 by CX-4945. Cell Signal..

[15] Götz C., Gratz A., Kucklaender U., Jose J. (2012). TF--a novel cell-permeable and selective inhibitor of human protein kinase CK2 induces apoptosis in the prostate cancer cell line LNCaP. Biochim. Biophys. Acta.

[16] Park J.H., Kim J.J., Bae Y.S. (2013). Involvement of PI3K-AKT-mTOR pathway in protein kinase CKII inhibition-mediated senescence in human colon cancer cells. Biochem. Biophys. Res. Commun..

[17] Dominguez I., Sonenshein G.E., Seldin D.C. (2009). Protein kinase CK2 in health and disease: CK2 and its role in Wnt and NF-kappaB signaling: linking development and cancer. Cell Mol. Life Sci..

[18] MacDonald B.T., Tamai K., He X. (2009). Wnt/beta-catenin signaling: components, mechanisms, and diseases. Dev. Cell.

[19] Tapia J.C., Torres V.A., Rodriguez D.A., Leyton L., Quest A.F. (2006). Casein kinase 2 (CK2) increases survivin expression via enhanced beta-catenin-T cell factor/lymphoid enhancer binding factor-dependent transcription. Proc. Natl. Acad. Sci. USA.

[20] Cozza G. (2017). The Development of CK2 Inhibitors: From Traditional Pharmacology to in Silico Rational Drug Design. Pharmaceuticals.

[21] Sarno S., Ruzzene M., Frascella P., Pagano M.A., Meggio F., Zambon A., Mazzorana M., Di Maira G., Lucchini V., Pinna L.A. (2005). Development and exploitation of CK2 inhibitors. Mol. Cell Biochem..

[22] Prudent R., Cochet C. (2009). New protein kinase CK2 inhibitors: jumping out of the catalytic box. Chem. Biol..

[23] Wilhelm N., Kostelnik K., Götz C., Montenarh M. (2012). Protein kinase CK2 is implicated in early steps of the differentiation of pre-adipocytes into adipocytes. Mol. Cell Biochem..

[24] Montenarh M. (2014). Protein kinase CK2 and angiogenesis. Adv. Clin. Exp. Med..

[25] St-Denis N.A., Litchfield D.W. (2009). Protein kinase CK2 in health and disease: From birth to death: the role of protein kinase CK2 in the regulation of cell proliferation and survival. Cell Mol. Life Sci..

[26] Al-Quobaili F., Montenarh M. (2012). CK2 and the regulation of the carbohydrate metabolism. Metabolism.

[27] Welker S., Götz C., Servas C., Laschke M.W., Menger M.D., Montenarh M. (2013). Glucose regulates protein kinase CK2 in pancreatic beta-cells and its interaction with PDX-1. Int. J. Biochem. Cell Biol..

[28] Wang K., Li F., Cui Y., Cui C., Cao Z., Xu K., Han S., Zhu P., Sun Y. (2019). The Association between Depression and Type 1 Diabetes Mellitus: Inflammatory Cytokines as Ferrymen in between?. Mediat. Inflamm..

[29] Andersson S.A., Olsson A.H., Esguerra J.L., Heimann E., Ladenvall C., Edlund A., Salehi A., Taneera J., Degerman E., Groop L. (2012). Reduced insulin secretion correlates with decreased expression of exocytotic genes in pancreatic islets from patients with type 2 diabetes. Mol. Cell. Endocrinol..

[30] Donath M.Y., Boni-Schnetzler M., Ellingsgaard H., Halban P.A., Ehses J.A. (2010). Cytokine production by islets in health and diabetes: cellular origin, regulation and function. Trends Endocrinol Metab..

[31] Oguntibeju O.O. (2019). Type 2 diabetes mellitus, oxidative stress and inflammation: examining the links. Int. J. Physiol. Pathophysiol. Pharm..

[32] Eldor R., Yeffet A., Baum K., Doviner V., Amar D., Ben-Neriah Y., Christofori G., Peled A., Carel J.C., Boitard C. (2006). Conditional and specific NF-kappaB blockade protects pancreatic beta cells from diabetogenic agents. Proc. Natl. Acad. Sci. USA.

[33] Wang D., Westerheide S.D., Hanson J.L., Baldwin Jr A.S. (2000). Tumor necrosis factor alpha-induced phosphorylation of RelA/p65 on Ser529 is controlled by casein kinase II. J. Biol. Chem..

[34] Ampofo E., Rudzitis-Auth J., Dahmke I.N., Roessler O.G., Thiel G., Montenarh M., Menger M.D., Laschke M.W. (2015). Inhibition of protein kinase CK2 suppresses tumor necrosis factor (TNF)-alpha-induced leukocyte-endothelial cell interaction. Biochim. Biophys. Acta.

[35] Bird T.A., Schooley K., Dower S.K., Hagen H., Virca G.D. (1997). Activation of nuclear transcription factor NF-kappaB by interleukin-1 is accompanied by casein kinase II-mediated phosphorylation of the p65 subunit. J. Biol. Chem..

[36] Jaksch C., Thams P. (2014). A critical role for CK2 in cytokine-induced activation of NFkappaB in pancreatic beta cell death. Endocrine.

[37] Duncan J.S., Turowec J.P., Vilk G., Li S.S., Gloor G.B., Litchfield D.W. (2010). Regulation of cell proliferation and survival: convergence of protein kinases and caspases. Biochim. Biophys. Acta.

[38] Meng R., Götz C., Montenarh M. (2010). The role of protein kinase CK2 in the regulation of the insulin production of pancreatic islets. Biochem. Biophys. Res. Commun..

[39] Spohrer S., Gross R., Nalbach L., Schwind L., Stumpf H., Menger M.D., Ampofo E., Montenarh M., Götz C. (2017). Functional interplay between the transcription factors USF1 and PDX-1 and protein kinase CK2 in pancreatic beta-cells. Sci. Rep..

[40] Zandomeni R., Bunick D., Ackerman S., Mittleman B., Weinmann R. (1983). Mechanism of action of DRB. III. Effect on specific in vitro initiation of transcription. J. Mol. Biol..

[41] Schneider C.C., Hessenauer A., Gotz C., Montenarh M. (2009). DMAT, an inhibitor of protein kinase CK2 induces reactive oxygen species and DNA double strand breaks. Oncol. Rep..

[42] Eizirik D.L., Miani M., Cardozo A.K. (2013). Signalling danger: endoplasmic reticulum stress and the unfolded protein response in pancreatic islet inflammation. Diabetologia.

[43] Tyka K., Jorns A., Turatsinze J.V., Eizirik D.L., Lenzen S., Gurgul-Convey E. (2019). MCPIP1 regulates the sensitivity of pancreatic beta-cells to cytokine toxicity. Cell Death Dis..

[44] Takai T., Matsuda T., Matsuura Y., Inoue K., Suzuki E., Kanno A., Kimura-Koyanagi M., Asahara S.I., Hatano N., Ogawa W. (2018). Casein kinase 2 phosphorylates and stabilizes C/EBPbeta in pancreatic beta cells. Biochem. Biophys. Res. Commun..

[45] Ampofo E., Sokolowsky T., Götz C., Montenarh M. (2013). Functional interaction of protein kinase CK2 and activating transcription factor 4 (ATF4), a key player in the cellular stress response. Biochim. Biophys. Acta.

[46] Schneider C.C., Ampofo E., Montenarh M. (2012). CK2 regulates ATF4 and CHOP transcription within the cellular stress response signalling pathway. Cell Signal..

[47] Papa F.R. (2012). Endoplasmic reticulum stress, pancreatic beta-cell degeneration, and diabetes. Cold Spring Harb. Perspect. Med..

[48] Oyadomari S., Koizumi A., Takeda K., Gotoh T., Akira S., Araki E., Mori M. (2002). Targeted disruption of the Chop gene delays endoplasmic reticulum stress-mediated diabetes. J. Clin. Investig..

[49] Jonsson J., Ahlgren U., Edlund T., Edlund H. (1995). IPF1, a homeodomain protein with a dual function in pancreas development. Int. J. Dev. Biol.

[50] McKinnon C.M., Docherty K. (2001). Pancreatic duodenal homeobox-1, PDX-1, a major regulator of beta cell identity and function. Diabetologia.

[51] Meng R., Al-Quobaili F., Müller I., Götz C., Thiel G., Montenarh M. (2010). CK2 phosphorylation of Pdx-1 regulates its transcription factor activity. Cell Mol. Life Sci..

[52] Claiborn K.C., Sachdeva M.M., Cannon C.E., Groff D.N., Singer J.D., Stoffers D.A. (2010). Pcif1 modulates Pdx1 protein stability and pancreatic beta cell function and survival in mice. J. Clin. Investig..

[53] Liu A., Oliver-Krasinski J., Stoffers D.A. (2006). Two conserved domains in PCIF1 mediate interaction with pancreatic transcription factor PDX-1. FEBS Lett..

[54] Klein S., Meng R., Montenarh M., Götz C. (2016). The Phosphorylation of PDX-1 by Protein Kinase CK2 Is Crucial for Its Stability. Pharmaceuticals.

[55] Ostertag M.S., Messias A.C., Sattler M., Popowicz G.M. (2019). The Structure of the SPOP-Pdx1 Interface Reveals Insights into the Phosphorylation-Dependent Binding Regulation. Structure.

[56] Melloul D., Tsur A., Zangen D. (2002). Pancreatic Duodenal Homeobox (PDX-1) in health and disease. J. Pediatric Endocrinol. Metab. JPEM.

[57] Lupp S., Götz C., Khadouma S., Horbach T., Dimova E.Y., Bohrer A.M., Kietzmann T., Montenarh M. (2014). The upstream stimulatory factor USF1 is regulated by protein kinase CK2 phosphorylation. Cell Signal..

[58] Frogne T., Sylvestersen K.B., Kubicek S., Nielsen M.L., Hecksher-Sorensen J. (2012). Pdx1 is post-translationally modified in vivo and serine 61 is the principal site of phosphorylation. PLoS ONE.

[59] An R., da Silva Xavier G., Semplici F., Vakhshouri S., Hao H.X., Rutter J., Pagano M.A., Meggio F., Pinna L.A., Rutter G.A. (2010). Pancreatic and duodenal homeobox 1 (PDX1) phosphorylation at serine-269 is HIPK2-dependent and affects PDX1 subnuclear localization. Biochem. Biophys. Res. Commun..

[60] Ardestani A., Paroni F., Azizi Z., Kaur S., Khobragade V., Yuan T., Frogne T., Tao W., Oberholzer J., Pattou F. (2014). MST1 is a key regulator of beta cell apoptosis and dysfunction in diabetes. Nat. Med..

[61] Ardestani A., Maedler K. (2016). MST1: a promising therapeutic target to restore functional beta cell mass in diabetes. Diabetologia.

[62] Servas C., Kiehlmeier S., Hach J., Gross R., Gotz C., Montenarh M. (2017). The mammalian STE20-like kinase 1 (MST1) is a substrate for the apoptosis inhibiting protein kinase CK2. Cell Signal..

[63] Komatsu M., Takei M., Ishii H., Sato Y. (2013). Glucose-stimulated insulin secretion: A newer perspective. J. Diabetes Investig..

[64] Hirokawa N. (1998). Kinesin and dynein superfamily proteins and the mechanism of organelle transport. Science.

[65] Donelan M.J., Morfini G., Julyan R., Sommers S., Hays L., Kajio H., Briaud I., Easom R.A., Molkentin J.D., Brady S.T. (2002). Ca2+-dependent dephosphorylation of kinesin heavy chain on beta-granules in pancreatic beta-cells. Implications for regulated beta-granule transport and insulin exocytosis. J. Biol. Chem..

[66] Gilon P., Henquin J.C. (2001). Mechanisms and physiological significance of the cholinergic control of pancreatic beta-cell function. Endocr. Rev..

[67] Budd D.C., McDonald J.E., Tobin A.B. (2000). Phosphorylation and regulation of a Gq/11-coupled receptor by casein kinase 1alpha. J. Biol. Chem..

[68] Torrecilla I., Spragg E.J., Poulin B., McWilliams P.J., Mistry S.C., Blaukat A., Tobin A.B. (2007). Phosphorylation and regulation of a G protein-coupled receptor by protein kinase CK2. J. Cell Biol..

[69] Luo J., Busillo J.M., Benovic J.L. (2008). M3 muscarinic acetylcholine receptor-mediated signaling is regulated by distinct mechanisms. Mol. Pharm..

[70] Willets J.M., Mistry R., Nahorski S.R., Challiss R.A. (2003). Specificity of g protein-coupled receptor kinase 6-mediated phosphorylation and regulation of single-cell m3 muscarinic acetylcholine receptor signaling. Mol. Pharm..

[71] Rossi M., Ruiz d.A.I., Barella L.F., Sakamoto W., Zhu L., Cui Y., Lu H., Rebholz H., Matschinsky F.M., Doliba N.M. (2015). CK2 acts as a potent negative regulator of receptor-mediated insulin release in vitro and in vivo. Proc. Natl. Acad. Sci. USA.

[72] Doliba N.M., Liu Q., Li C., Chen P., Liu C., Naji A., Matschinsky F.M. (2017). Inhibition of Cholinergic Potentiation of Insulin Secretion from Pancreatic Islets by Chronic Elevation of Glucose and Fatty Acids: Protection by Casein Kinase 2 Inhibitor. Mol. Metab..

[73] Cantley J., Davenport A., Vetterli L., Nemes N.J., Whitworth P.T., Boslem E., Thai L.M., Mellett N., Meikle P.J., Hoehn K.L. (2019). Disruption of beta cell acetyl-CoA carboxylase-1 in mice impairs insulin secretion and beta cell mass. Diabetologia.

[74] Ronnebaum S.M., Joseph J.W., Ilkayeva O., Burgess S.C., Lu D., Becker T.C., Sherry A.D., Newgard C.B. (2008). Chronic suppression of acetyl-CoA carboxylase 1 in beta-cells impairs insulin secretion via inhibition of glucose rather than lipid metabolism. J. Biol. Chem..

[75] Imai Y., Cousins R.S., Liu S., Phelps B.M., Promes J.A. (2019). Connecting pancreatic islet lipid metabolism with insulin secretion and the development of type 2 diabetes. Ann. N. Y. Acad. Sci..

[76] Zhang S., Kim K.H. (1997). Protein kinase CK2 down-regulates glucose-activated expression of the acetyl-CoA carboxylase gene. Arch. Biochem. Biophys..

[77] Armstrong S.A., Barry D.A., Leggett R.W., Mueller C.R. (1997). Casein kinase II-mediated phosphorylation of the C terminus of Sp1 decreases its DNA binding activity. J. Biol. Chem..

[78] Schuster D.P. (2010). Obesity and the development of type 2 diabetes: the effects of fatty tissue inflammation. Diabetes Metab. Syndr. Obes. Targets Ther..

[79] Cho H.J., Park J., Lee H.W., Lee Y.S., Kim J.B. (2004). Regulation of adipocyte differentiation and insulin action with rapamycin. Biochem. Biophys. Res. Commun..

[80] Klemm D.J., Leitner J.W., Watson P., Nesterova A., Reusch J.E., Goalstone M.L., Draznin B. (2001). Insulin-induced adipocyte differentiation. Activation of CREB rescues adipogenesis from the arrest caused by inhibition of prenylation. J. Biol. Chem..

[81] Wabitsch M., Hauner H., Heinze E., Teller W.M. (1995). The role of growth hormone/insulin-like growth factors in adipocyte differentiation. Metabolism.

[82] Schwind L., Nalbach L., Zimmer A.D., Kostelnik K.B., Menegatti J., Grasser F., Gotz C., Montenarh M. (2017). Quinalizarin inhibits adipogenesis through down-regulation of transcription factors and microRNA modulation. Biochim. Et Biophys. Acta. Gen. Subj..

[83] Chen Q., Hao W., Xiao C., Wang R., Xu X., Lu H., Chen W., Deng C.X. (2017). SIRT6 Is Essential for Adipocyte Differentiation by Regulating Mitotic Clonal Expansion. Cell Rep..

[84] Schäfer B., Götz C., Dudek J., Hessenauer A., Matti U., Montenarh M. (2009). KIF5C, a new binding partner for protein kinase CK2 with a preference for CK2à’. Cell Mol. Life Sci..

[85] Schäfer B., Götz C., Montenarh M. (2008). The kinesin I family member KIF5C is a novel substrate for protein kinase CK2. Biochem. Biophys. Res. Commun..

[86] Bae J.S., Park S.H., Jamiyandorj U., Kim K.M., Noh S.J., Kim J.R., Park H.J., Kwon K.S., Jung S.H., Park H.S. (2016). CK2alpha/CSNK2A1 Phosphorylates SIRT6 and Is Involved in the Progression of Breast Carcinoma and Predicts Shorter Survival of Diagnosed Patients. Am. J. Pathol..

[87] Borgo C., Milan G., Favaretto F., Stasi F., Fabris R., Salizzato V., Cesaro L., Belligoli A., Sanna M., Foletto M. (2017). CK2 modulates adipocyte insulin-signaling and is up-regulated in human obesity. Sci. Rep..

[88] Scherer P.E. (2019). The many secret lives of adipocytes: implications for diabetes. Diabetologia.

[89] Nakamura A., Miyoshi H., Ukawa S., Nakamura K., Nakagawa T., Terauchi Y., Tamakoshi A., Atsumi T. (2018). Serum adiponectin and insulin secretion: A direct or inverse association?. J. Diabetes Investig..

[90] Okamoto M., Ohara-Imaizumi M., Kubota N., Hashimoto S., Eto K., Kanno T., Kubota T., Wakui M., Nagai R., Noda M. (2008). Adiponectin induces insulin secretion in vitro and in vivo at a low glucose concentration. Diabetologia.

[91] Heiker J.T., Wottawah C.M., Juhl C., Kosel D., Morl K., Beck-Sickinger A.G. (2009). Protein kinase CK2 interacts with adiponectin receptor 1 and participates in adiponectin signaling. Cell Signal..

[92] Juhl C., Morl K., Beck-Sickinger A.G. (2011). Adiponectin receptor 1 interacts with both subunits of protein kinase CK2. Mol. Cell Biochem..

[93] Arfken C.L., Reno P.L., Santiago J.V., Klein R. (1998). Development of proliferative diabetic retinopathy in African-Americans and whites with type 1 diabetes. Diabetes Care.

[94] Frank R.N. (2004). Diabetic retinopathy. N. Engl. J. Med..

[95] Gariano R.F., Gardner T.W. (2005). Retinal angiogenesis in development and disease. Nature.

[96] Zhou B., Ritt D.A., Morrison D.K., Der C.J., Cox A.D. (2016). Protein Kinase CK2alpha Maintains Extracellular Signal-regulated Kinase (ERK) Activity in a CK2alpha Kinase-independent Manner to Promote Resistance to Inhibitors of RAF and MEK but Not ERK in BRAF Mutant Melanoma. J. Biol. Chem..

[97] Ruzzene M., Bertacchini J., Toker A., Marmiroli S. (2017). Cross-talk between the CK2 and AKT signaling pathways in cancer. Adv. Biol. Regul..

[98] Feng D., Welker S., Korbel C., Rudzitis-Auth J., Menger M.D., Montenarh M., Laschke M.W. (2012). Protein kinase CK2 is a regulator of angiogenesis in endometriotic lesions. Angiogenesis.

[99] Siddiqui-Jain A., Drygin D., Streiner N., Chua P., Pierre F., O’Brien S.E., Bliesath J., Omori M., Huser N., Ho C. (2010). CX-4945, an orally bioavailable selective inhibitor of protein kinase CK2, inhibits prosurvival and angiogenic signaling and exhibits antitumor efficacy. Cancer Res..

[100] Baxter R.C. (2001). Signalling pathways involved in antiproliferative effects of IGFBP-3: a review. Mol. Pathol. MP.

[101] Jiang Y., Zhang Q., Steinle J.J. (2014). Intravitreal injection of IGFBP-3 restores normal insulin signaling in diabetic rat retina. PLoS ONE.

[102] Joussen A.M., Poulaki V., Le M.L., Koizumi K., Esser C., Janicki H., Schraermeyer U., Kociok N., Fauser S., Kirchhof B. (2004). A central role for inflammation in the pathogenesis of diabetic retinopathy. FASEB J..

[103] Joussen A.M., Doehmen S., Le M.L., Koizumi K., Radetzky S., Krohne T.U., Poulaki V., Semkova I., Kociok N. (2009). TNF-alpha mediated apoptosis plays an important role in the development of early diabetic retinopathy and long-term histopathological alterations. Mol. Vis..

[104] Zhang Q., Soderland D., Steinle J.J. (2014). TNFalpha inhibits IGFBP-3 through activation of p38alpha and casein kinase 2 in human retinal endothelial cells. PLoS ONE.

[105] Ljubimov A.V., Caballero S., Aoki A.M., Pinna L.A., Grant M.B., Castellon R. (2004). Involvement of protein kinase CK2 in angiogenesis and retinal neovascularization. Invest. Ophthalmol Vis. Sci.

[106] Kramerov A.A., Saghizadeh M., Pan H., Kabosova A., Montenarh M., Ahmed K., Penn J.S., Chan C.K., Hinton D.R., Grant M.B. (2006). Expression of protein kinase CK2 in astroglial cells of normal and neovascularized retina. Am. J. Pathol..

[107] Ampofo E., Schmitt B.M., Laschke M.W., Menger M.D. (2018). Function of protein kinase CK2 in thrombus formation. Platelets.

[108] Welters H.J., Kulkarni R.N. (2008). Wnt signaling: relevance to beta-cell biology and diabetes. Trends Endocrinol. Metab. TEM.

[109] Rulifson I.C., Karnik S.K., Heiser P.W., ten Berge D., Chen H., Gu X., Taketo M.M., Nusse R., Hebrok M., Kim S.K. (2007). Wnt signaling regulates pancreatic beta cell proliferation. Proc. Natl. Acad. Sci. USA.

[110] Chen Y., Hu Y., Zhou T., Zhou K.K., Mott R., Wu M., Boulton M., Lyons T.J., Gao G., Ma J.X. (2009). Activation of the Wnt pathway plays a pathogenic role in diabetic retinopathy in humans and animal models. Am. J. Pathol..

